# Repeatability of the macular pigment spatial profile: A comparison of objective versus subjective classification

**DOI:** 10.1111/aos.13725

**Published:** 2018-08-29

**Authors:** Irene Ctori, Omar A. Mahroo, Katie M. Williams, Christopher J. Hammond, Byki Huntjens

**Affiliations:** ^1^ Applied Vision Research Centre City, University of London Northampton Square London UK; ^2^ Department of Twin Research and Genetic Epidemiology King's College London St Thomas’ Hospital Campus London UK; ^3^ Department of Ophthalmology King's College London St Thomas’ Hospital Campus London UK; ^4^ Retinal Service Moorfields Eye Hospital London UK; ^5^ UCL Institute of Ophthalmology London UK

**Keywords:** fundus autofluorescence, heritability, macular pigment optical density, repeatability, spatial profile

## Abstract

**Purpose:**

Classification of macular pigment (MP) spatial profile phenotypes varies and is often based on subjective visualisation. We investigated repeatability of MP optical density (MPOD) comparing an objective versus subjective profiling system.

**Methods:**

The coefficient of repeatability (CoR) was calculated for point MPOD values (0–3.8°) obtained by dual‐wavelength fundus autofluorescence (FAF) from two scans obtained in a single visit of 40 healthy individuals (39 ± 9 years). For each individual's dataset, the MP profile was classified as exponential, ring‐like or central dip using an objective method (based on deviations away from an exponential fit), as well as by subjective visual profiling. Existing FAF images of 88 monozygotic (MZ) and 69 dizygotic (DZ) twin pairs were reanalysed using the objective profiling method and concordance and heritability of ring‐like profiles determined.

**Results:**

The CoR was 0.23 at 0° and 0.06 at 0.8°. Agreement of objective profiling between scans was excellent (*κ* = 0.85, 95% CI 0.69 to 1.00; p < 0.0005). Subjective profiling showed moderate agreement between scans (*κ* = 0.48, 95% CI 0.23 to 0.73; p < 0.0005). Agreement between objective and subjective classification was low (*κ* = 0.23, 95% CI 0.04 to 0.42; p = 0.02). Concordance for the ring‐like profile using objective profiling was 0.74 for MZ compared to 0.36 for DZ twins. Heritability was calculated as 81.5% (95% confidence interval 61.1–93.1%).

**Conclusion:**

Compared to visual assessment, objective MP profiling is a more reliable method and should be considered in future observational and interventional studies. In addition, MP profile phenotypes showed high heritability.

## Introduction

Macular pigment (MP) is thought to serve a protective function shielding the central photoreceptors from the damaging effects of blue light (Junghans et al. 2001; Barker et al. 2011). The amount of MP, measured as macular pigment optical density (MPOD) and its density distribution across the macula, that is its spatial profile, varies among healthy individuals (Iannaccone et al. 2007; Wolf‐Schnurrbusch et al. 2007; Nolan et al. 2008). Various approaches at averaging the MPOD across an area of the retina have been presented, as it has been suggested that a single central MPOD measurement is a poor predictor of the total amount of MP present (Robson et al. 2003). Averaged MPOD calculated from MPOD measured at several eccentricities from 0.25° to 3° has been reported (Nolan et al. 2008). Alternatively, the area under the exponential curve fit to a subject's MP spatial distribution data has been calculated based on MPOD data obtained by heterochromatic flicker photometry, HFP (Nolan et al. 2008; Kirby et al. 2010) and by two‐wavelength FAF (Hammond et al. 2012). This integrated MPOD value provides information as to the overall quantity of MP presents across the macula as opposed to measurements at a single retinal eccentricity. This is a useful indicator to consider as it has been shown that the overall amount of MP present varies according to its density distribution pattern (Trieschmann et al. 2003).

The spatial profile phenotype of MP across the retina may play a role in the protection of the eye against age‐related macular degeneration (AMD) (Trieschmann et al. 2003). In particular, the presence of an annulus of increased MPOD was reported to be three times less common in eyes with the presence of AMD compared to healthy eyes (Dietzel et al. 2011). Although the different techniques for measuring MPOD may cause inconsistencies when comparing data between studies (Delori et al. 2001; Canovas et al. 2010), the presence of a ring‐like structure or a secondary peak within the MP spatial profile has been demonstrated by HFP (Hammond et al. 1997; Kirby et al. 2009, 2010) and also by imaging using two‐wavelength FAF (Berendschot & van Norren 2006; Delori et al. 2006; Dietzel et al. 2011; Tariq et al. 2014). However, the classification of spatial profile phenotypes varies across the literature and there is currently no universal consensus on a single classification system. Various techniques have been described including objective analysis of secondary maxima–minima pairs (Delori et al. 2006) and mathematical analysis of a combination of an exponential and Gaussian fit to the data distribution (Berendschot & van Norren 2006). Quantification analysis of MP derived from FAF images has been used to characterize different MP spatial profile phenotypes (Trieschmann et al. 2003), as has subjective visual assessment of two‐wavelength FAF scan images (Tariq et al. 2014). As well as inconsistencies with nomenclature, classification of a ring‐like spatial profile may be affected by measurement error, noise in the data or a product of an artefact of the MPOD measurement method (Delori 2004). While it has been shown that repeatability of point MPOD measurements is dependent on the instrument employed (Snodderly et al. 2004; Tang et al. 2004; de Kinkelder et al. 2010), test–retest repeatability of MPOD measurements is often carried out only at a single 0.5° location for HFP methods (Snodderly et al. 2004; Tang et al. 2004; Bartlett et al. 2010; de Kinkelder et al. 2010; Iannaccone et al. 2016) or at 0.5° and 2° eccentricity using two‐wavelength FAF (Trieschmann et al. 2006). If MP spatial profile classification is based on deviations from an exponential fit to the data (Berendschot & van Norren 2006; Nolan et al. 2008; Huntjens et al. 2014; Ctori & Huntjens 2017) or an increase relative to central MPOD (Nolan et al. 2012), it is important to consider the reliability of the MPOD measurement not only according to the instrument used but also the repeatability of the MPOD measurement at each of the different retinal eccentricities tested.

The aim of this study was to investigate the repeatability of individual point MPOD measurements (between 0° and 4° retinal eccentricities) using two‐wavelength FAF imaging. In addition, we investigated the agreement of spatial profile phenotype obtained between scans using an objective classification method as well as a visual classification method. The intervisit agreement of the objective classification method has not previously been described. We also determined the heritability of spatial profiles as established by objective profiling and compared this with the previously obtained estimate using subjective profile assessment. Finally, we tested the hypothesis that a single central MPOD measure is a poor predictor of the amount of MP present.

## Patients and Methods

### Two‐wavelength FAF imaging for heritability study

Two‐wavelength FAF imaging was carried out as part of a twin heritability study described in detail elsewhere (Hammond et al. 2012) that included 314 healthy Caucasian female twin volunteers (aged 16–50 years) recruited from the TwinsUK registry at St Thomas’ Hospital (London, UK) (Liew et al. 2005). All participants had healthy retinas and clear crystalline lenses. In brief, following mydriasis, two‐wavelength FAF imaging was performed on both eyes of each participant using a modified confocal scanning laser ophthalmoscope (Heidelberg Engineering, Heidelberg, Germany) providing high‐resolution images at 488 and 514 nm wavelengths. The intensity of a greyscale map, generated by digital subtraction of the images obtained at the two wavelengths, was proportional to the MPOD at each retinal location. The instrument's software generates a plot of MPOD against eccentricity by averaging MPOD measurements at each retinal location in concentric rings according to the distance from the foveal centre (Wustemeyer et al. 2003). The right eye of each twin was included in the analysis.

### Repeatability study

Approval to reanalyse the previously acquired two‐wavelength FAF images (Hammond et al. 2012) was obtained from the TwinsUK Resource Executive Committee of St Thomas’ Hospital, London. Data analysis for this study took place at the Department of Twin Research and Genetic Epidemiology, Kings College London, St Thomas’ Hospital Campus, London. Subjects that had two scans on the same eye taken within a single visit were eligible for inclusion into the repeatability study.

### Analysis of FAF images

The MPOD profile for each subject was generated using the automated ‘find fovea’ function available within the instrument's software. This was performed as an attempt to have a consistent approach to locating the fovea (Sasamoto et al. 2010) rather than manually placing the cursor at the perceived centre of the fovea. Point MPOD values at 0°, 0.1°, 0.8°, 1.8°, 2.8° and 3.8° were extracted from the instrument's software for each scan. Eccentricity values were selected on the basis of a previous investigation by our research group (Huntjens et al. 2014; Ctori & Huntjens 2017) in which MPOD was measured by HFP at predefined eccentricities at 0°, 0.8°, 1.8°, 2.8° and 3.8° (with the average of MPOD at 6.8° and 7.8° used as the reference value).

#### Classification of MP spatial profile phenotypes

A MP spatial profile phenotype was assigned to the MPOD data for each FAF scan based on the method detailed in a previous investigation (Huntjens et al. 2014). For objective profile classification, the MPOD values at 0°, 0.1°, 0.8°, 1.8°, 2.8° and 3.8° were plotted against retinal eccentricity for each subject. An exponential curve was fitted to the MPOD data up to 3.8° allowing the exponential function to float, rather than assuming a fixed negligible value at the peripheral reference location (Putnam & Bassi 2015; Ctori & Huntjens 2017). An exponential profile was assigned if the measured MPOD value at 0°, 0.8° and 1.8° was within one coefficient of repeatability (CoR) (i.e. the average within‐subject SD) of the value predicted by the fitted exponential curve (Table 2). Profiles with MPOD values deviating greater than one CoR above the exponential fit at 0.8° were assigned a ring‐like classification (Hammond et al. 1997). It has been shown that ring‐like structures occur at approximately 0.7–0.8° eccentricity from the fovea as determined by HFP (Hammond et al. 1997; Kirby et al. 2009; Huntjens et al. 2014) or FAF methods (Hammond et al. 1997; Delori et al. 2006). A deviation more than one CoR below the expected value at 0° was classified as a central dip (Huntjens et al. 2014; Ctori & Huntjens 2017). For subjective classification, experienced investigator (OM) visually inspected both FAF images of each participant for the presence of a ring‐like pattern or central dip as described elsewhere (Tariq et al. 2014). Each FAF image was inspected in a random order, blind to the results of the first scan and blind to the objective classification.

#### Intervisit agreement of MP spatial profile phenotypes

Agreement of MP spatial profile phenotype between scans obtained in a single visit using objective classification (Huntjens et al. 2014) versus a subjective visual classification method (Dietzel et al. 2011; Tariq et al. 2014) was determined.

### Heritability study

Concordance of the ring‐like profile for monozygotic (MZ) and dizygotic (DZ) twin pairs was determined from previously obtained two‐wavelength FAF data for 157 twin pairs (Hammond et al. 2012). The heritability of spatial profiles as established by objective profiling was quantified and compared with the previously obtained estimate using subjective profile assessment.

#### Calculation of integrated MPOD

It has been shown that an exponential function describes the MP spatial profile well (Hammond et al. 1997). A measure of the integrated MPOD (MPODint) based on the area under the MPOD distribution curve was calculated by integrating the area under the best fit curve. This was calculated for each participant's MPOD data set using the MPOD values at 0°, 0.8°, 1.8°, 2.8° and 3.8°. The trapezium rule was used in a two‐dimensional coordinate system to calculate the area under the curve from 0° to 3.8° (i.e. MPODint), based on the same approach detailed by Kirby et al. (2010). We tested the hypothesis that a single central MPOD measure is a poor predictor of the overall amount of MP present, with MPODint used as a proxy.

### Statistical analysis

All statistical analyses were performed using spss version 22.0 for Windows (spss Inc., Chicago, USA). Values in the text and tables are presented as mean ± standard deviation (SD). MPOD measurements are in log units. The CoR was calculated as CoR = 1.96s, where s is the SD of the difference between pairs of MPOD measurements between visits one and two (Bland & Altman 1986). Limits of agreement (LoA) were determined as the mean difference between pairs of MPOD measurements ± CoR. The LoA indicate the range within which 95% of the differences between measurements will lie (Bland & Altman 1986, 1999; McAlinden et al. 2011). Agreement of classification of the MP spatial profile was evaluated by the overall percentage of agreement between visits or scans and by the Kappa measure of agreement, κ (Landis & Koch 1977; Sim & Wright 2005). Case‐wise concordance for the presence of a ring‐like or central dip profile was calculated separately for MZ and DZ twins as 2*C*/(2*C* + *D*), where *C* is the number of twin pairs concordant and *D* the number discordant (Tariq et al. 2014). Heritability calculation for pigment profiles was performed as described previously (Tariq et al. 2014) with maximum likelihood structural equation twin modelling, using the OpenMx package (http://openmx.psyc.virginia.edu) in R (http://www.r-project.org).

## Results

The demographics of the participants included in the repeatability and heritability studies are presented in Table 1.

**Table 1:** Demographics of the participants included in the repeatability and heritability studies

	Number of participants	Number of eyes	Age (mean ± SD, years)	Gender	Ethnicity
Repeatability study	40	40	39 ± 8.6	Female	Caucasian
Heritability study	314	314	39 ± 8.8	Female	Caucasian

### Repeatability study

**Figure 1:**
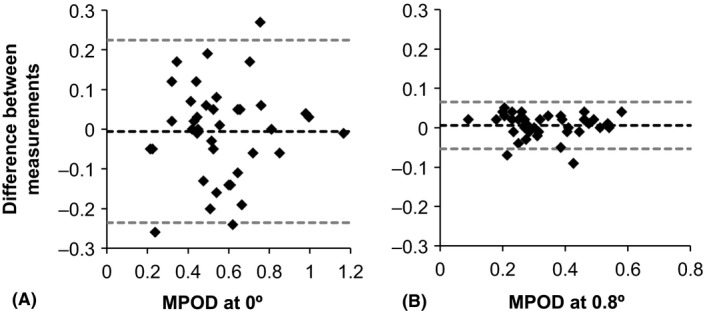
Bland–Altman plots to show repeatability of MPOD measurements. Plots show the difference in MPOD measurements between scans (A) at 0° and (B) at 0.8°. Black dashed line represents the mean of the two measurements. Grey dashed lines indicate the upper and lower limits of agreement indicating the range within which 95% of the differences between measurements are expected to lie.

Mean MPOD at 0° was 0.57 ± 0.22 for the first scan and 0.57 ± 0.21 for the second (*t*(39) = −0.18, p = 0.86), with a CoR of 0.23 calculated. At 0.1°, the CoR was 0.15 and reduced to ≤0.06 from 0.8° and beyond (Table 2). Bland–Altman plots for the 0° and 0.8° locations are shown in Fig. 1.

**Table 2:** Mean MPOD ± SD for the repeatability study (*n* = 40). Repeatability measures according to the retinal eccentricity measured are also displayed

Retinal Eccentricity (°)	Mean ± SD Scan 1	Mean ± SD Scan 2	CoR	Variance (%)
0	0.57 ± 0.22	0.57 ± 0.21	0.23	4.8
0.1	0.55 ± 0.21	0.54 ± 0.19	0.15	4.6
0.8	0.34 ± 0.12	0.33 ± 0.12	0.06	1.4
1.8	0.10 ± 0.04	0.09 ± 0.04	0.04	0.2
2.8	0.05 ± 0.02	0.05 ± 0.02	0.03	0.05
3.8	0.04 ± 0.01	0.03 ± 0.02	0.03	0.02

#### Repeatability of spatial profiling

**Figure 2:**
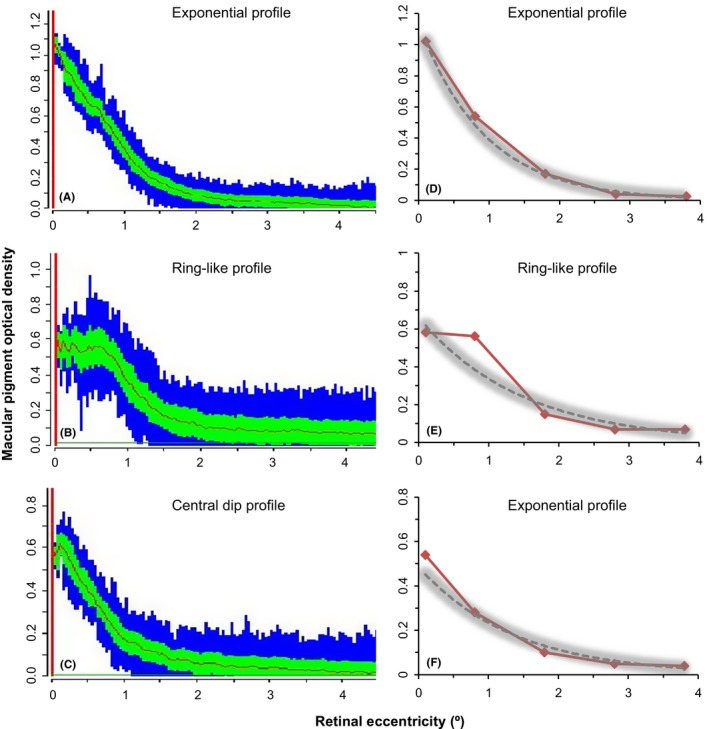
(A–C) Examples of the three MP spatial profile phenotypes (exponential profile, ring‐like profile or central dip profile) as classified by visual inspection of fundus autofluorescence. (D–F) shows the profiles following objective classification. MPOD (red line) along the *y*‐axis (extracted from the FAF data on the left‐hand side) plotted against retinal eccentricity along the *x*‐axis. The shaded grey area schematically represents one CoR above and below the exponential fit to the data (grey dashed line).

Examples of spatial profiles are shown in Fig. 2. FAF images were *visually* classified as an exponential (A), ring‐like (B) or central dip (C) profile. The data were extrapolated to complete objective classification as described in the methods (Fig. 2D‐F). According to the objective profile classification, (D) shows an exponential profile; (E) shows a ring‐like profile (whereby MPOD at 0.8° is more than 1 CoR above the exponential fit line) and (F) shows that while image (C) was visually classified as a central dip, central MPOD is not more than 1 CoR below the exponential fit line and is therefore *objectively* classified as an exponential profile.

The frequency distribution of the three different spatial profile types is presented in Table 3. Overall percentage of agreement of objective classification of the MP spatial profile between each pair of FAF scans was 93% with a *κ*‐value of 0.85 (95% confidence interval 0.69–1.00, p < 0.0005). Subjective visual classification of the MP spatial profile between FAF scans resulted in 73% overall percentage of agreement, and a *κ*‐value of 0.48 (95% confidence interval 0.23–0.73, p < 0.0005). The agreement between the objective and subjective classification for all 80 FAF scans resulted in overall percentage agreement of 60%, with a *κ*‐value of 0.23 (95% confidence interval 0.04 to 0.42, p = 0.02).

**Table 3:** Frequency of MP spatial profile types determined by objective and subjective classification (n = 40). Results presented as %, with the actual number in brackets

	Exponential		Ring‐like		Central dip	
	%	(*n*)	%	(*n*)	%	(*n*)
Objective spatial profiling						
Scan 1	52.5	(21)	47.5	(19)	0	(0)
Scan 2	60.0	(24)	40.0	(16)	0	(0)
Subjective visual profiling						
Scan 1	62.5	(25)	30	(12)	7.5	(3)
Scan 2	57.5	(23)	35	(14)	7.5	(3)

### Heritability study

Objective classification of MP spatial profile phenotype identified 71% as exponential and 29% as ring‐like profiles. There were no central dip profiles identified by the objective classification method. Case‐wise concordance for the ring‐like profile using objective methods was calculated as 0.74 for MZ and 0.36 for DZ twins. Formal calculation of heritability of the ring‐like macular pigment profile as established by the objective methods yielded an estimate of 81.5% of the variance being attributable to additive genetic factors (95% confidence interval 61.1–93.1%).

According to visual subjective profiling, 64% presented with an exponential profile while 27% had ring‐like and 9% central dip profiles. Case‐wise concordance for subjective profile classification resulted in 0.80 for MZ and 0.41 for DZ twins.

#### Correlation between single point MPOD and integrated MPOD

The correlation between single point measures of MPOD with MPODint (0–3.8) was calculated to test how well a single measure of MPOD describes the overall amount of MP present (Table 4).

**Table 4:** Correlation of MPOD at single central eccentricities with integrated MPODint (0–3.8°) among the participants of the twin study (*n* = 314). P < 0.0005 for all

	Mean	SD	Correlation with MPODint (0–3.8), *R* ^2^
MPOD at 0.1°	0.58	0.18	0.64
MPOD at 0.8°	0.34	0.13	0.90
MPOD at 1.8°	0.10	0.05	0.84
MPODint (0–3.8)	0.67	0.24	n/a

## Discussion

### Repeatability study

The results of the present investigation demonstrate a large variation in the CoR for central MPOD measurements quantified from FAF scans (Table 2). Feasibility of MP quantification using a grey scale analysis of FAF images obtained from two different instruments (HRA2 and S3300 Spectralis HRA‐OCT; Heidelberg Engineering, Heidelberg, Germany) was evaluated in an investigation including 34 normal subjects (Delori et al. 2011). Several technical modifications were suggested to reduce measurement errors, including implementing new alignment software as well as correction of the data to compensate for the absorption of the ocular media. Although such a correction algorithm was not applied to the absolute measure of MPOD in the current study, the prevalence of significant lens opacity in the study cohort would be expected to be low given the average age of the participants. In addition, the incident radiation would be absorbed similarly between 0° and 3.8° eccentricity and is therefore unlikely to be a significant factor in computations of relative MPOD. We propose that the poor CoR at 0° is most likely to be due to the algorithm used by the inbuilt ‘find fovea’ software, possibly because the fovea is defined according to a single pixel and therefore more likely influenced by noise. Nevertheless, there was a good within‐session repeatability of around 0.05 optical density units from 0.1° and beyond, consistent with previous investigations (Trieschmann et al. 2006; Delori et al. 2011). These findings indicate that measurement error has little influence on MPOD measurements quantified from the two‐wavelength FAF imaging technique employed in the current study at eccentricities beyond 0°. That being said, we propose that several (more than 2) measurements of MPOD may be needed to ensure robust values (Loughman 2010).

#### Repeatability of spatial profiling

Our results show that the methods employed for measurement and classification of the MP spatial profile are robust to test–retest variability. This is in accordance with a previous study based on sixteen individuals in which the profile type was shown to persist on repeated testing (Kirby et al. 2009). The objective method of classification of the MP spatial profile used in the current investigation is based on deviations away from an exponential fit to the data distribution taking into account the measurement error of the instrument according to the location at which MPOD is being measured (Huntjens et al. 2014). The intervisit agreement of this classification method has not previously been described. There was excellent repeatability (93%; *κ* = 0.85, p < 0.0005) of profiling by objective analysis; whereas repeatability of profiling by subjective visual analysis between scans was lower (73%; *κ* = 0.48; p < 0.0005). This finding implies that the objective method is a more reliable method of MP spatial profiling. Notably, in the original twin study (Tariq et al. 2014), the kappa measure of agreement between two graders of ‘ring versus no‐ring’ was reported as 0.705 (p‐value not given), illustrating that although this is a fast method, variability could arise with subjective profiling classification. Furthermore, our results indicate poor agreement between the objective and subjective classification method applied to the same FAF scan image (60%; *κ* = 0.23, p = 0.02), illustrating how the same data can give rise to different phenotype classifications depending on the classification system that is applied. Indeed, while only three profiles were identified as central dips by visual inspection in the present study (Fig. 2C), objectively, these deviations were smaller than the CoR and therefore classified objectively as exponential (Fig. 2F). This, in turn, highlights the difficulties in comparing studies that have employed different classification techniques.

### Heritability study

Although it is possible to extract MPOD values from FAF scans corresponding to several retinal eccentricities between 0° and 0.8°, this is a time‐consuming task when performed manually. Nonetheless, among the 314 twin participants, prevalence of the ring‐like profile (29%) determined objectively compares well with the 26% reported in the original study (Tariq et al. 2014). This suggests that ring‐like MP structures can be identified by objective classification based on the limited eccentricities used in our analysis. In the present investigation, a consistent method to identify the central MPOD measurement utilizing the ‘find fovea’ function of the Heidelberg software was incorporated, whereas the original study (Tariq et al. 2014) had identified the centre of the scan as the location where MPOD was maximal. Despite this variation in methodology, case‐wise concordance of nonexponential MP profiles was 0.74 for MZ twins; approximately double that for DZ twins based on our objective MP profiling. This is in accordance with the original study in which it was shown that there was greater concordance of a ring‐like profile in MZ compared to DZ twins (Tariq et al. 2014). Similarly, in the present study, the objective profiling method yielded a similar high estimate of heritability, 81.5% (95% CI, 61.1–93.1%), compared to heritability for the ring‐like profile determined by subjective visual assessment of 84.0% (95% CI, 63.7–96.4%) (Tariq et al. 2014) confirming that genetic factors appear to be important in determining spatial pigment profiles.

#### Correlation between single point MPOD and integrated MPOD

We calculated the MPODint (0–3.8) and used this value as a proxy for the overall amount of MP. It should be noted that the central 3.8° is likely to underestimate the total complement of MP, as a substantial component is located beyond this eccentricity (low MPOD but a large area in comparison with the central macula) (Degli Esposti et al. 2012). Nonetheless, although there was a strong correlation between the central MPOD measurement and MPODint, this relationship was significantly stronger for single MPOD measurements at 0.8° (Table 4). The advantage of an integrated measure is that it allows comparisons to be drawn between individuals regardless of the MP spatial distribution phenotype. We propose that an integrated measure is a better indicator of the amount of MP present as opposed to a single central MPOD measurement and may be a more appropriate parameter to report in future studies.

### Limitations

Using the automated ‘find fovea’ feature may lead to an inaccurate determination of 0° eccentricity. An investigation on the difference between finding the fovea manually versus automatically would be of benefit. It is worth noting that the FAF scans generated in this investigation were not available to view in 3D. Further investigation of classification based on 3D profiles would be of interest. According to the objective classification method, none of the 314 twins showed a central dip. A limitation of the present study is the definition of the different spatial profiles. Until an agreed system has been established, the varying peak widths, including broad‐shaped MP without a central peak and ring‐shaped patterns, could be considered instead (Elsner et al. 1998; Sharifzadeh et al. 2006, 2008). While our study focused on the distribution of macular pigment in a healthy cohort, MP profiles of unhealthy eyes (for example in AMD or in macular telangiectasia type 2) should be considered in future studies.

## Conclusion

The repeatability of single MPOD measurements varies according to retinal eccentricity. Based on the two‐wavelength FAF technique, MPOD fluctuations greater than 0.23 at 0°, 0.12 at 0.1° and 0.06 at 0.8° can be considered as clinically significant perturbations in the data as opposed to instrument noise. Therefore, taking several measurements of MPOD may be needed to ensure robust values. Our findings demonstrate that applying an objective classification system provides a reliable method of MP spatial profiling that is robust to test–retest variability. Although currently there are limitations in obtaining this output manually from FAF scans, automated quantification of MP spatial profiles may serve in future as a powerful objective parameter of macular structure, which may be a useful biomarker in epidemiological studies and also in understanding what factors may confer protection or vulnerability to macular diseases. In addition, we confirmed that genetic factors appear to be important in determining MP profile phenotypes.
